# Microarray Analysis of Cell Cycle Gene Expression in Adult Human Corneal Endothelial Cells

**DOI:** 10.1371/journal.pone.0094349

**Published:** 2014-04-18

**Authors:** Binh Minh Ha Thi, Nelly Campolmi, Zhiguo He, Aurélien Pipparelli, Chloé Manissolle, Jean-Yves Thuret, Simone Piselli, Fabien Forest, Michel Peoc'h, Olivier Garraud, Philippe Gain, Gilles Thuret

**Affiliations:** 1 Laboratory for corneal graft biology, engineering and imaging', EA2521, SFR143, Faculty of Medicine of the University Jean Monnet, Saint-Etienne, France; 2 Department of Ophthalmology, University Hospital, Saint-Etienne, France; 3 Department of Pathology, University Hospital, Saint-Etienne, France; 4 Service de Biologie Intégrative et de Génétique Moléculaire (SBIGeM), Institut de biologie et de technologies de Saclay (iBiTec-S), Commissariat à l'Energie Atomique, Gif-sur-Yvette, France; 5 Eye Bank, Etablissement Français du Sang Loire/Auvergne, Saint-Etienne, France; 6 Institut Universitaire de France, Paris, France; Instituto Butantan, Brazil

## Abstract

Corneal endothelial cells (ECs) form a monolayer that controls the hydration of the cornea and thus its transparency. Their almost nil proliferative status in humans is responsible, in several frequent diseases, for cell pool attrition that leads to irreversible corneal clouding. To screen for candidate genes involved in cell cycle arrest, we studied human ECs subjected to various environments thought to induce different proliferative profiles compared to ECs in vivo. Donor corneas (a few hours after death), organ-cultured (OC) corneas, in vitro confluent and non-confluent primary cultures, and an immortalized EC line were compared to healthy ECs retrieved in the first minutes of corneal grafts. Transcriptional profiles were compared using a cDNA array of 112 key genes of the cell cycle and analysed using Gene Ontology classification; cluster analysis and gene map presentation of the cell cycle regulation pathway were performed by GenMAPP. Results were validated using qRT-PCR on 11 selected genes. We found several transcripts of proteins implicated in cell cycle arrest and not previously reported in human ECs. Early G1-phase arrest effectors and multiple DNA damage-induced cell cycle arrest-associated transcripts were found in vivo and over-represented in OC and in vitro ECs. Though highly proliferative, immortalized ECs also exhibited overexpression of transcripts implicated in cell cycle arrest. These new effectors likely explain the stress-induced premature senescence that characterizes human adult ECs. They are potential targets for triggering and controlling EC proliferation with a view to increasing the cell pool of stored corneas or facilitating mass EC culture for bioengineered endothelial grafts.

## Introduction

The corneal endothelium, which maintains stable corneal transparency in humans, is essential to visual-system performance [1]. It is a monolayer of hexagonal, densely packed corneal endothelial cells (ECs) separating the corneal stroma from the aqueous humor. By actively regulating hydration of the stroma, it prevents the onset of edema which, by disorganizing the collagen fibrils, would impair the passage of light [2]. In humans, corneal ECs lose their proliferative ability during fetal development [3], [4] and are consequently vulnerable in vivo. If the endothelium sustains a lesion, its integrity, which is necessary for its function, is only maintained by the migration and enlargement of the ECs adjacent to the lesion, without mitosis. As a result, when endothelial cell density (ECD) falls below a critical threshold (which depends on the type, extent and kinetics of the pathological process), irreversible corneal edema sets in. Endothelial diseases are a frequent cause of corneal blindness, for which only a corneal graft can restore vision. The graft, whether full thickness (penetrating keratoplasty, PKP) or endothelial (endothelial keratoplasty, EK), supplies a new pool of functional ECs from the donor cornea. However, after both types of graft, ECD falls rapidly in the first 6 months, then more slowly, but at a higher rate than the physiological EC loss rate of 0.6% a year [5]. Recipients thus frequently need more than one graft during their lifetime.

The absence of corneal EC division is therefore responsible for significant corneal blindness worldwide. Knowing which cellular mechanisms are implicated in human corneal EC cycle arrest would thus allow the development of new therapeutic tools to trigger and control EC proliferation. In vivo, ECs are blocked in G1 phase but maintain a residual proliferative capacity that can be exploited in vitro. The senescent state of central ECs in vivo may result from many simultaneous mechanisms (exposed in [6], [7], [8]): low level of growth factors in aqueous humor, lack of autocrine stimulation by growth factors synthesized by ECs, cell cycle entry inhibition by TGF-β2 present in aqueous humor, contact inhibition induced by formation of mature cell-cell and cell-substrate junctions, oxidative DNA damage resulting in a permanently high level of mRNA or proteins of the cyclin-dependent kinase inhibitors (CDKI) p27, p21, and p16, and cascades of blocking points for G1-S transition, especially belonging to the p53 pathway.

There are at least three possible areas of development for advanced therapy medicinal products in the field of ECs: 1/Ex vivo enrichment of grafts in EC is a realistic prospect [9] that would improve both the quality (prolonged survival in recipients) and the quantity of available graft tissue (by upgrading corneas whose ECD was initially too low). 2/In vitro mass culture of ECs would also allow bioengineering of endothelial graft tissue. 3/In parallel, it would become conceivable to treat early stages of primitive (Fuch's) or secondary endothelial dystrophies in vivo by injecting ECs in the anterior chamber [10]. Until now, three main approaches were used to trigger human EC proliferation (Table 1): 1/A general approach of improving culture conditions to limit cell death and promote cell cycle stimulating factors. 2/A focused approach focusing on a limited number of key cycle-controlling proteins considered as EC cycle blocking points. 3/Selecting and stimulating potential EC progenitor or stem cells located at the extreme periphery of the endothelium [11], [12].

**Table 1:** Published experimental methods for triggering human corneal endothelial cell proliferation.

Approach		Principle	Biological model	Author, year
FOCUSED	Immortalization of primary HCEC	Transduction of **simian virus 40 large T-antigen** expression	human (in vitro)	Wilson 1993 [92], Feldman 1993 [93]
		Transformation with papillomavirus **E6/E7** oncogenes	human (in vitro)	Wilson 1995 [94]
		Retroviral transduction of **Cdk4R24C/CyclinD1** to human corneal endothelial	human (in vitro)	Yokoi 2012 [75]
		Transduction of human **telomerase reverse transcriptase expression** (hTERT)	human (in vitro)	Liu 2012 [95]
		Transduction of **hTERT** combined with **p53** downregulation or **CDK4** overexpression	human (in vitro)	Sheerin 2012 [8]
	E2F2 overexpression	Transduction of **E2F2**	human (ex vivo)	McAlister 2005 [96]
	CDKI down regulation	siRNA **p27Kip1**	human (in vitro)	Kikuchi 2006 [25]
		Electroporation with **p21+p16** siRNA	human (in vitro)	Joyce 2010 [97]
	Anti-apoptotic effect	Transduction of baculoviral **p35** or mammalian **Bcl-xL** for anti-apoptotic gene therapy	human (in vitro)	Fuchsluger 2011 [98]
GENERAL	Medium optimization	Organ culture at 32°C with 8% FCS	human (ex vivo)	Slettedal 2008 [15]
		Evaluation of 4 basic culture **medias** (DMEM, OptiMEM-I, DMEM/F12, Ham's F12/M199) in isolation & propagation	human (in vitro)	Peh 2011 [99]
		Reduction of reactive oxygen species by **rapamycin** & **cysteamine**	human (in vitro)	Shin 2012 [100]
	Culture support	**Decellularized bovine corneal posterior lamellae** as carrier matrix for cultivated cells	human & bovin (ex vivo, in vitro)	Bayyoud 2012 [101]
		**Gelatin hydrogel carrier** sheet for corneal endothelial transplantation.	human (in vitro)	Watanabe 2011 [102]
	Cell-cell contact release	**EDTA**+organ culture at 37°C+10% FCS†, 10 ng/mL EGF†, 20 ng/mL FGF†	human (ex vivo)	Senoo 2000 [103], Patel 2009 [104]
		**Localized lesion**+organ culture at 37°C+10% FCS†, 10 ng/mL EGF†, 20 ng/mL bFGF†	human (ex vivo)	Senoo, 2000 [105]
REGENERATION	HCEC precursor	Isolation of **HCEC precursors i**n vitro by sphere-forming assay	human (in vitro)	Yokoo 2005 [106]
	Stromal cornea-derived precursors	Differentiation of **stromal cornea-derived precursors** to functional corneal endothelium by cell isolation in medium containing retinoic acid, GSK 3b inhibitor and Rock inhibitor Y-27632	human (in vitro)	Hatou 2012 [107]
	Trabecular meshwork stem-like cell	Local progenitors for the corneal endothelium and **trabecular meshwork** personalized stem cell therapy in corneal endothelial diseases and glaucoma (Review)	/	Yu 2012 [108]

† FCS: Fœtal calf serum; EGF: Epidermal growth factor; FGF: Fibroblast growth factor.

In the present study, we used a specific chip of 112 genes implicated in cell cycle regulation to examine human ECs subjected to six environments thought to induce different proliferative profiles: in vivo, post mortem (PM), organ culture (OC) stored corneas, confluent primary culture (CPC), non-confluent primary culture (NCPC), and immortalized EC line (ECL). We hypothesized that these six conditions form a coherent gradation from the well-recognized non-proliferative status of ECs in vivo to the immortalized ECs line that continuously proliferates, with four intermediate states liable to allow identification of differentially expressed genes. Unlike short-term cold storage, OC in fetal calf serum (FCS) supplemented medium maintains EC viability and functionality for up to 5 weeks. OC is not known to promote EC proliferation, although it facilitates endothelial wound healing by migration of cells in the vicinity of local defects in the endothelial layer [13]. Only OC with 8% FCS, a method that remains an exception [14], seems likely to promote cell mitosis [15]. It was thus important to determine whether routine OC induces significant transcriptional profile changes liable to reveal the stimulation of certain ECs with residual proliferative capacity. In vitro, primary cultures of ECs with FCS and growth factors usually have limited proliferative capacity and rapidly become senescent, especially when reaching confluence. Conversely, ECLs obtained by transducing simian virus 40 large T-antigene expression display very high proliferative capacity [16].

Hence, the crossing analysis of these models, from in vivo to the ECL, helps us to understand mechanisms implicated in EC division and senescence, thus giving new potential key points for triggering mitosis. Gottsch's major work on human endothelial cornea genomics proposed an analysis of all transcripts present in normal endothelia [17] and in Fuch's dystrophy [18] by using an ultra-sensitive SAGE method and Affymetrix HU95a microarrays containing about 12000 genes previously characterized in terms of function or disease association. In addition, Gottsch's team created a comprehensive database of human corneal gene expression. To date, no focused analysis has been done on the expression of genes implicated in cell cycle control in human ECs.

## Materials and Methods

### Ethics statement

All procedures conformed to the tenets of the Declaration of Helsinki for biomedical research involving human subjects. Central corneal buttons removed as waste during surgery were collected as per the usual protocol in force in our University Hospital and by presumed consent further to the written information given to all admitted patients. This protocol was written by our Hospital's commission for clinical research and innovation and accepted by the local ethics committee (CPP Sud Est I, University Hospital, Saint Etienne, France). Corneas assigned to scientific use were procured from bodies donated to science (Laboratory of Anatomy, Faculty of Medicine) as permitted by French law. Each donor volunteers their body and gives written consent to the Laboratory of Anatomy; no extra specific approval by the ethics committee is required

### Materials

Serum-free medium (OptiMEM-1), Petri dish, 6-well tissue culture plates, T25 flasks and fetal bovine serum (FBS) were purchased from Fisher Bioblock Scientific (Vaulx-Milieu, France). Medium 199 (M199), epidermal growth factor (EGF; from mouse submaxillary glands), nerve growth factor (NGF; from mouse submaxillary glands), bovine pituitary extract (also known as Keratinocyte Growth Supplement), ascorbic acid, calcium chloride, chondroitin sulphate, 0.02% EDTA solution (EDTA disodium salt), antibiotic-antimycotic solution, gentamicin, trypsin, glutamine, penicillin-streptomycin solution, insulin and RNA loading dye were purchased from Sigma-Aldrich (Saint-Quentin Fallavier, France). Cell attachment reagent (FNC Coating Mix) was purchased from Gentaur (Brussels, Belgium). RNeasy mini Kit, RNase-free DNase I and Taq PCR Master Mix were purchased from Qiagen (Courtaboeuf, France). Oligo(dT)_12–18_ primer, dNTP Mix (10 mM), Ribonuclease H, dithiothreitol (DTT; 0.1 M), 5× First-Strand Buffer, SuperScript II Reverse Transcriptase, Ribosomal protein S27a (RPS27A) forward and reverse primers were purchased from Invitrogen (Cergy Pontoise, France). Biotin-16-UTP was purchased from Roche Applied Science (Meylan, France). Cell Cycle OligoArray (Oligo GEArray Human Cell Cycle OHS-020) and Oligo GEArray Starter Kit (SABiosciences Corporation, SuperArray, Frederick, MD) were purchased from Tebu-bio (Le Perray en Yvelines, France). Inventoried TaqMan Gene Expression Assays and TaqMan Universal PCR Master Mix were purchased from Applied Biosystems (Courtaboeuf, France).

### Human corneal tissue

In order to study ECs in the physiological in vivo state as closely as possible, ECs from patients undergoing corneal graft for keratoconus were chosen. Keratoconus is a common non-inflammatory, progressive disease in which the endothelial layer is healthy and not known to be involved in the disease pathogenesis. Penetrating keratoplasty consists of replacing the central full-thickness cornea by a normal donor cornea. Consequently, a normal endothelium can be obtained during surgery. Eight central corneas (diameter 8.25 mm) were obtained during penetrating grafts for keratoconus, immediately after trephination (in vivo group). ECs were collected as explained above. Time from trephination to endothelial freezing was about 20 minutes. Thirty-four supplementary human corneas assigned to scientific use (procured from bodies donated to science) were used in this study. Donor and procurement characteristics are presented in Table 2. Except for the in vivo group, corneas were procured by a 16–18 mm diameter in situ excision, as per the procedure recommended in France for corneas intended for transplantation. Twelve corneas, forming the post-mortem group, were immediately used. Eleven corneas, forming the OC group, were immediately placed in 100 mL of OC medium (Corneamax®, Eurobio, Les Ulis, France) at 31°C in a dry incubator. Five corneas were used to carry out confluent primary culture (CPC group). The last six corneas were used to carry out non-confluent primary culture (NCPC group). SV40 immortalized ECs (HCECT-12) were bought from DMSZ (Braunschweig, Germany) [16], [19] and are henceforth referred to as the endothelial cell line (ECL).

**Table 2:** Characteristics of donor corneas.

Condition	Biological replicate	Age (years)	Death/retrieval time (hours)	Storage time (days)
In vivo	15	44 (27–81)	/	/
Post-mortem	12	79 (49–98)	12 h25 (4 h–21 h45)	/
Organ culture	11	69 (49–85)	11 h40 (5 h30–15 h55)	16 (13–24)
Confluent primary culture	5	78 (65–88)	13 h08 (6 h30–21 h25)	13 (8–18)
Non-confluent primary culture	6	78 (58–89)	15 h26 (11 h–16 h30)	21 (4–48)

Values were expressed as median (min-max).

### Cell culture

#### Primary culture

ECs were isolated and cultured according to published protocols [20], [21], [22], [23], [24], [25]. Briefly, Descemet membrane and ECs were peeled off under an operating microscope and separated into small fragments that were immersed in culture medium containing OptiMEM-I, 10% FBS, 5 ng/mL EGF, 20 ng/mL NGF, 100 µg/mL pituitary extract, 20 µg/mL ascorbic acid, 200 mg/L calcium chloride, 0.08% chondroitin sulphate, 50 µg/mL gentamicin, and antibiotic/antimycotic solution diluted 1/100. Isolated cells and pieces of Descemet membrane that still contained attached cells were plated in six-well tissue culture plates precoated with undiluted FNC Coating Mix. Cultures were then incubated at 37°C in a 5% carbon dioxide, humidified atmosphere. Medium was changed every 2 days. Confluent primary cultured cells were then trypsinized, resuspended in culture medium, and subcultured into T25 flasks that had been precoated and grown to 50% or 100% confluence.

#### Immortalized cell line

The ECL was cultured as previously described [26] with some technical changes. Briefly, cells were grown in T25 flasks with culture medium F99 (1∶2 dilution of medium 199 and Ham's F12) supplemented with 0.3% chondroitin sulphate, 2% glutamine, 0.5% penicillin-streptomycin solution, 0.02% ascorbic acid, 0.02% insulin and 10% FCS. The cultures were maintained in 5% CO_2_ at 37°C. Culture medium was changed every 2–3 days.

### RNA extraction

Under an operating microscope, Descemet membrane with endothelium was peeled off from the underlying stroma with forceps to avoid contamination by other cell types. Two corneas were pooled for the OC and post-mortem groups (whole cornea), and four for the in vivo group (8 mm diameter trephination). Microarrays were consequently performed in duplicate for each condition. This approach minimizes inter individual differences and was extensively published [27], [28], [29]. Non-confluent and confluent primary cultures and ECL cultures were trypsinized and pelleted. Descemet membrane fragments and ECs were then frozen at −80°C until RNA isolation. Total RNA was isolated using an RNeasy mini kit as per the manufacturer's instructions. Before further use, total RNA was treated with RNase-free DNase I [30]. After extraction, RNA was checked for stability and DNA contamination by running a sample with an RNA loading dye on a 1% agarose gel, and visualization was done by staining with ethidium bromide. Lack of genomic DNA contamination was confirmed by polymerase chain reaction (PCR) amplification of RNA samples that had not been reverse-transcribed. RNA quantity was determined by spectrophotometric absorbance of the sample at 260 nm measurement, and purity was determined based on the ratio at 260 nm to that of 280 nm (A_260_/A_280_) using a BioMate 3 Series spectrophotometer (Thermo Fisher Scientific, Cergy Pontoise, France). Only RNA samples with A_260_/A_280_ ratio >1.8 were used for further experiments. Samples were frozen at −80°C until use in microarrays and qRT-PCR.

### Polymerase chain reaction

PCR was carried out on RNA of each sample in order to check possible contamination with genomic DNA. Amplification was performed by the standard method on Ribosomal protein S27a (RPS27A) gene using Taq PCR Master Mix as per the manufacturer's instructions. RPS27A is a component of the 40S subunit of the ribosome, and has been previously identified as a valid control gene in expression studies conducted among human malignant and control tissues [31], [32]. PCRs were performed in 100 µl reactions using 300 ng RNA, 2.5 units of Taq DNA Polymerase, 1× PCR Buffer (containing 1.5 mM MgCl_2_), 200 µM of each dNTP and 0.5 µM of each primer. The sequences of human RPS27A primers, described elsewhere [33], [34], were as follows: forward primer, 5′-TCGTGGTGGTGCTAAGAAAA-3′; and reverse primer, 5′-TCTCGACGAAGGCGACTAAT-3′. Amplifications were carried out using the following cycling parameters: initial denaturation at 95°C for 10 minutes, denaturation at 95°C for 60 seconds, annealing at 53°C for 60 seconds, and extension at 72°C for 60 seconds. PCR amplification was done for 40 cycles with a final extension at 72°C for 10 minutes. RPS27A cDNA from cell line, obtained after RT, was used as a positive control. For a negative control, PCR was run using PCR reaction mixture with primers but no nucleic acid. Following amplification, the PCR products were characterized in 1% agarose-ethidium bromide gels in Trisacetate buffer. A product was obtained from PCR performed on positive control but not on RNAs and negative control, indicating that samples were not contaminated by genomic DNA (data not shown).

### Reverse transcription

First-strand cDNA synthesis was carried out on 300 ng of total RNA in a final volume of 20 µl with SuperScript II Reverse Transcriptase as per the manufacturer's protocol. Briefly, after addition in nuclease-free microcentrifuge tubes of 300 ng of total RNA, 0.1 µl Oligo(dT)_12–18_ (500 µg/ml), 1 µl dNTP Mix (10 mM each) and sterile distilled water to complete the volume at 12 µl, the mixture was heated at 65°C for 5 minutes. 4 µl of 5× First-Strand Buffer and 2 µl of DTT were then added and the mix incubated at 42°C for 2 minutes. Incubation at 42°C for 50 minutes was performed after the addition of 1 µl of SuperScript II RT. The reaction was inactivated by heating at 70°C for 15 minutes. To remove RNA complementary to the cDNA, 1 µl of *E. coli* RNase H (two units) was added and the mixture incubated at 37°C for 20 minutes and then chilled on ice. cDNA were stored at −20°C until use in qPCR.

### GEArray

Expression levels of cell cycle-specific genes were examined by human cell cycle Oligo GEArray (OHS-020). The Oligo GEArray Human cell cycle Microarray profiled the expression of 112 key genes involved in cell cycle regulation, such as cyclin-dependent kinases (CDKs) involved in cell cycle progression and the proteins that regulate them (cyclins, CDK inhibitors, CDK phophatases and kinases). Genes essential for DNA damage and mitotic spindle checkpoints and genes in the SCF (*CUL1*, *SKP2*) and APC (anaphase promoting complex, with *CDC16-20*, *ANAPC2-4-5*) ubiquitin-conjugation complexes were also included. Microarrays were performed in two biological replicates for each group. Briefly, as described elsewhere [35], [36], [37], [38], [39], using the TrueLabeling-AMP 2.0 kit, 1 µg of purified total RNA was reversely transcribed to obtain cDNA and converted into biotin-labeled cRNA using biotin-16-UTP (Roche) by in vitro transcription. The biotin-labeled cRNA was purified using the cRNA clean-up kit. Prehybridization (2 hours) and hybridization (overnight) was done at 60°C in a hybridization oven using 3 µg purified labeled cRNA target as per manufacturer's protocol. After washing and blocking the array membranes, alkaline phosphatase-conjugated streptavidin was allowed to bind and CDP-Star substrate chemiluminescence was detected by exposure to x-ray film. The film was acquired using a 16-bit desktop scanner and saved as a grayscale 16-bit TIFF file. Absence of signal saturation (number of grey levels <255) was verified for each image. Array data were analyzed using the web-based GEArray Expression Analysis Suite software (SuperArray Bioscience). All signal intensities were background subtracted (subtraction of minimum value), and normalized to the housekeeping genes (RPS27A; GAPDH; B2M; HSP90AB1 and ACTB). For each set of duplicates, the mean value for each gene was determined and used to calculate the fold-changes: the in vivo group served as reference sample. The cut-off induction determining expression was ≥1.5 fold changes [40], [41], [42], (fold change = normalized result of groups/normalized result of the control group). Genes suiting both the above criteria were considered to be upregulated or downregulated with biological relevance. To gather reliable data, careful quality control of experiments, data extraction and standardization approaches were maintained throughout the experimental process.

#### Gene ontology (GO) and cluster analysis

The enriched biological processes GO terms for each cell cycle phase was determined by uploading the gene list on the Database for Annotation, Visualization, and Integrated Discovery (DAVID) version 6.7, 2013 (http://david.abcc.ncifcrf.gov/ [43], [44], (Table S1 in File S1). Clustering of genes expression by cell cycle phase was conducted with the software R version 2.15.0, 2012 [45]. The fold-change expression values were represented in logarithmic scale in heatmap graph.

#### Pathway profile analysis

Gene Map Annotator and Pathway Profiler (GenMAPP) version 2.1 was used to analyze GEArrays results. Fold-change expression values of each biological group versus the in vivo group were related to the Kyoto Encyclopedia of Genes and Genomes (KEGG) pathway of human cell cycle [46], [47]. Fold-change absolute values over 1.5 were considered as upregulated or downregulated and classified in three classes: 1.5 to 5 folds, 5 to 10 folds, and 10 folds and more.

### Real-time quantitative PCR

To confirm the cDNA arrays findings, real-time quantitative PCR were carried out on 11 selected genes: Cyclin D1; Cyclin E1; p21; p27; p16; p15; GADD45A, DIRAS3, culin 1, MKI67 and PCNA using an AB 7500 Fast Real-Time PCR System (Applied Biosystems). Assays were performed with TaqMan Universal PCR Master Mix and Inventoried Taqman Gene Expression Assays (Table 3) as per the manufacturer's instructions. Briefly, in wells of a 0.2 ml optical-grade 96-well PCR plate, we placed a 25 µl reaction mixture of 12.5 µl TaqMan Universal PCR Master Mix, 900 nM of each unlabeled primers (forward and reverse), 250 nM TaqMan MGB (minor groove binder) probe and 25 ng cDNA for each of the target genes. The assay's probes incorporated a 5′ reporter dye (6-carboxy-fluorescein, 6-FAM™dye label) and a 3′ non-fluorescent quencher (NFQ) and spanned an exon junction (Table 3): genomic DNA could not be detected. The thermal cycle conditions, as previously described [48] and as per the manufacturer's instructions, consisted of AmpErase activation (50°C, 2 min) and Taq activation (95°C, 10 min), followed by 40 cycles of denaturation (95°C, 15 sec), annealing and elongation (60°C, 1 min). The AB 7500 system measures a fluorescent accumulation of PCR product by continuous monitoring. 18S rRNA was used as an endogenous control to normalize for variation in the amount of cDNA template. Each sample also consisted of two biological replicates and three technical replicates. No Template Controls (NTC: PCR mixture without cDNA) were performed to rule out cross contamination of reagents, on each target as well as on endogenous control. All data were analyzed using the 7500 Fast System Detection Software version 1.2.3 (Applied Biosystems). After normalization of measurements using the endogenous control, results were analyzed using the comparative critical threshold (ΔΔCT) method [49], [50], [51] to compare differences between samples. As for the microarray, the in vivo group served as reference.

**Table 3:** TaqMan gene expression assays for quantitative real-time PCR used to validate microarray results on a subset of 11 genes of interest.

Assay ID	NCBI Gene Reference	Gene Symbol	Gene Name	Target Exons	Amplicon Length
Hs01026536_m1	NM_001238.1	*CCNE1*	Cyclin E1	11–12	64
Hs00277039_m1	NM_053056.2	*CCND1*	Cyclin D1	2–3	94
Hs00696862_m1	NM_002592.2	*PCNA*	Proliferating cell nuclear antigen	1–2	95
Hs01032443_m1	NM_002417.4	*MKI67*	Antigen identified by monoclonal antibody Ki-67	8–9	66
Hs00190723_m1	NM_004675.2	*DIRAS3*	DIRAS family, GTP-binding RAS-like 3	1–2	115
Hs00169255_m1	NM_001199741.1	*GADD45A*	Growth arrest and DNA-damage-inducible, alpha	2–3	123
	NM_001924.3			3–4	123
Hs00355782_m1	NM_078467.1	*CDKN1A*	Cyclin-dependent kinase inhibitor 1A (p21^Cip1^)	2–3	66
Hs00153277_m1	NM_004064.3	*CDKN1B*	Cyclin-dependent kinase inhibitor 1B (p27^Kip1^)	1–2	71
Hs00233365_m1	NM_058195.2	*CDKN2A*	Cyclin-dependent kinase inhibitor 2A (p16^Ink4A^)	2–3	117
Hs00365249 m1	NM_078487.2	*CDKN2B*	Cyclin-dependent kinase inhibitor 2B (p15^INK4B^)	1–2	128
Hs00269187_m1	NM_003592.2	*CUL1*	Cullin 1	2–3	165
Hs99999901_s1	X03205.1	*18S*	Eukaryotic 18S rRNA (Endogenous Controls)	___	187

### Statistical analysis

Spearman's correlation coefficient (r) was calculated between the biological replicate of each group studied by microarray, in order to estimate reproducibility, and between microarray and qRT-PCR results to estimate the reliability of microarrays. *P*<0.05 was considered statistically significant.

## Results

### Gene expression profiles in endothelial cells from different environments

**Figure 1:**
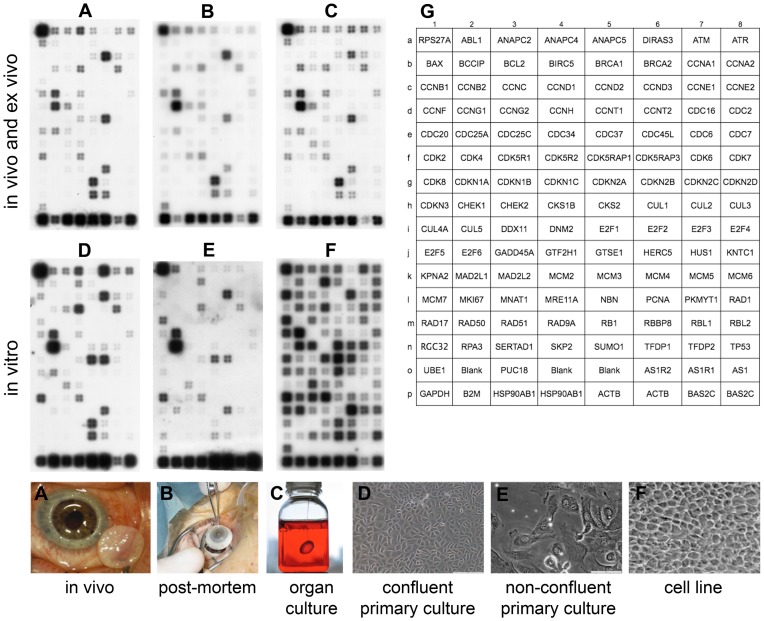
Microarray analysis of mRNA extracted from endothelial cells collected on six biological models. (**A**) in vivo corneas (reference group); (**B**) post-mortem corneas; (**C**) organ-cultured corneas; (**D**) confluent primary culture; (**E**) non-confluent primary culture; and (**F**) in vitro confluent cell line. Genes expressed were identified as a specific hybridization signal that appeared as tetra-spots. In addition to the 112 genes of interest, each chip contained five housekeeping genes for normalization (GADPH, beta-2-microglobuline (B2M), 2× HSP90AB1, 2× Actin beta (ACTB)), seven negative controls: PUC18 plasmid DNA, Artificial Sequence 1 related 2 (AS1R2), Artificial Sequence 1 Related 1 (AS1R1), Artificial Sequence 1 (AS1) and three blank spots, and two positive detection controls: two biotinylated Artificial Sequence 2 Complementary sequence (BAS2C) at increasing gradient. A key to gene coordinates is shown in panel **G**.

Detection of cell cycle gene transcripts by GEArray was successful for the six types of EC (Fig. 1). For each EC type, normalized values of biological duplicates were concordant with Spearman's correlation coefficients between biological replicates respectively of 0.891 for in vivo, 0.881 for PM, 0.972 for OC, 0.974 for CPC, 0.792 for NCPC, and 0.981 for the ECL (p<0.001 for each).

**Figure 2:**
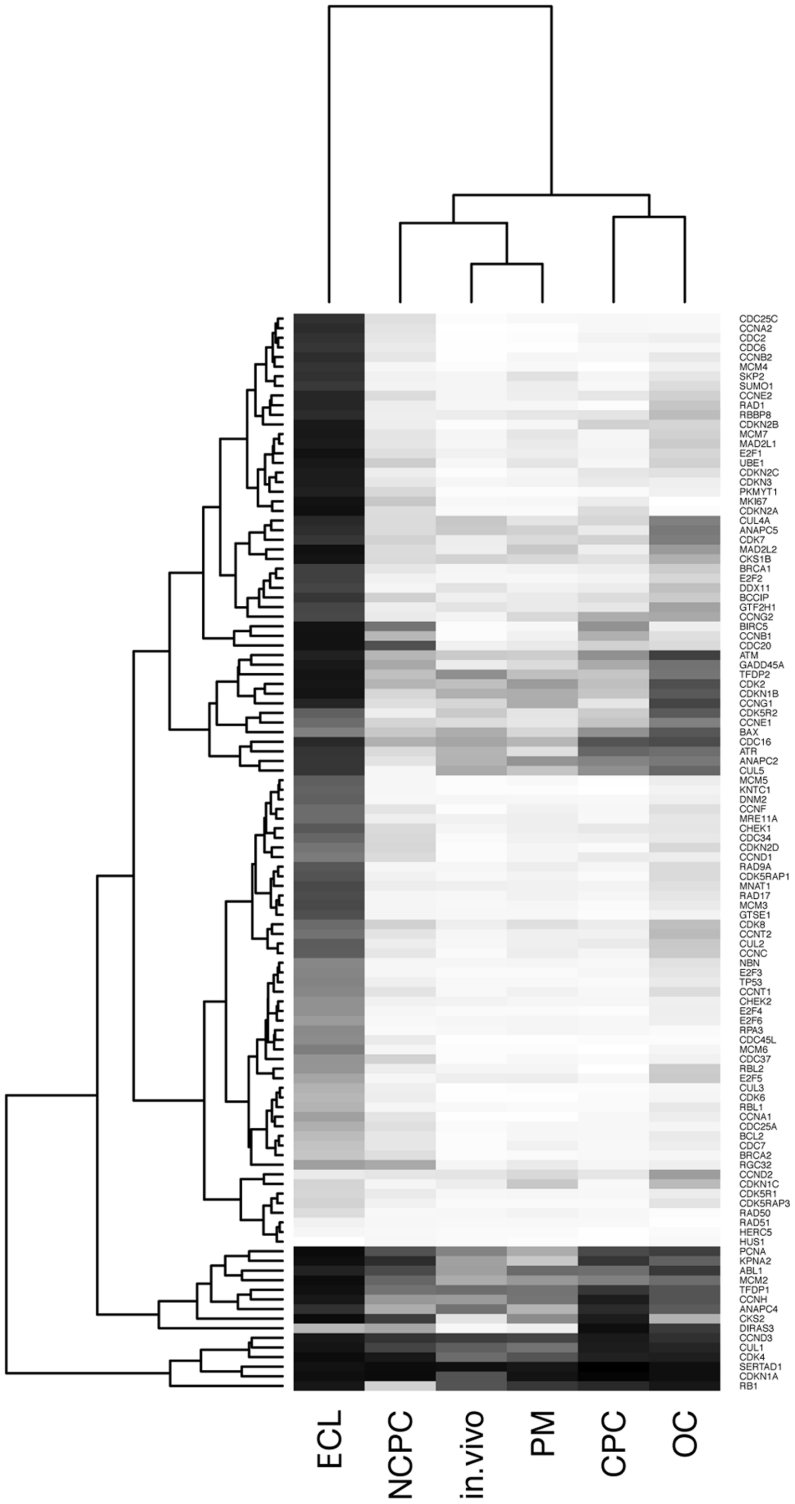
Clustergram analysis of expression of 112 cell cycle genes in endothelial cells of the six different biological models: in vivo, post mortem (PM), organ culture (OC), in vitro confluent primary culture (CPC), in vitro non confluent primary culture (NCPC), and cell line (ECL). Normalized expressions of all models were represented on the heatmap graph. Gray scale from 0 (blank) to 1 (black) represents variation between minimum and maximum normalized values.

Using cluster analysis of normalized values of the 112 cell cycle gene expressions, the heatmap representation (Fig. 2) exhibited the highest similarity between cell cycle gene expression of EC in vivo and PM. Samples collected from donors with short death to retrieval time (under 24 h) still had the same properties as the in vivo samples. OC shares similar patterns of expression with CPC and NCPC. The ECL clearly had a completely different profile, with activation of most cell cycle genes, and could consequently be considered a positive control for EC proliferation.

**Figure 3:**
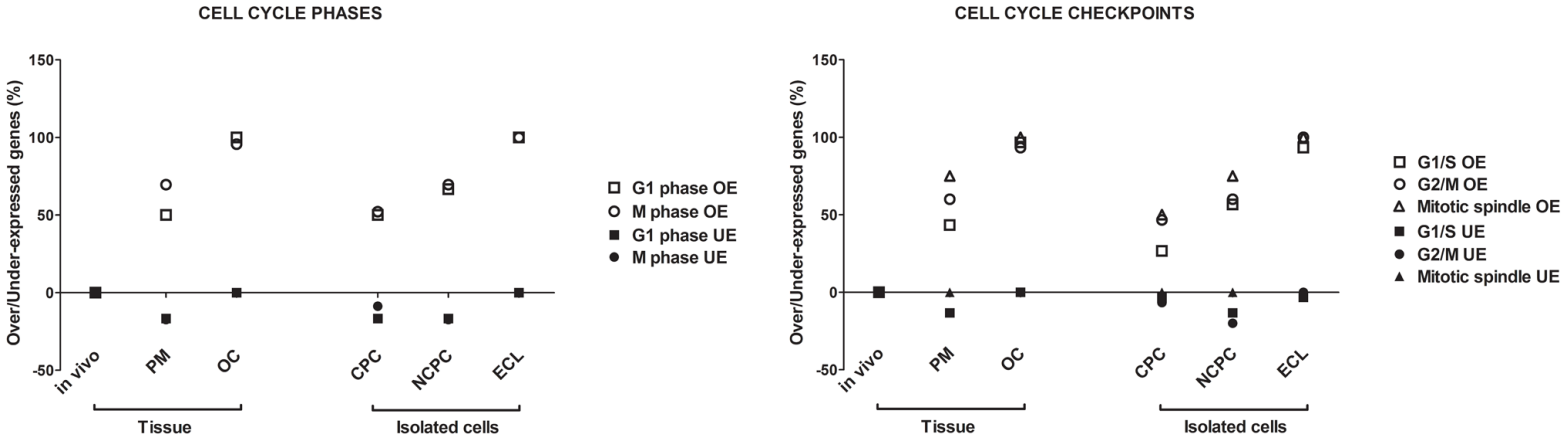
Variations of genes implicated in cell cycle phases and checkpoints. Percentage of genes overexpressed (OE) or underexpressed (UE) compared to in vivo condition (fold-change ≥1.5) in ECs of the different biological models: post mortem (PM), organ culture (OC), confluent primary culture (CPC), non-confluent primary culture (NCPC), and endothelial cell line (ECL). S and G2-phase genes (n = 4, see Table S1 in File S1), were not shown.

Based on GO classification, 62 of 112 genes analyzed by GEArray were directly classified in cell cycle phases and checkpoints; 40 other genes were not classified. The percentage of genes over- or underexpressed is presented in Figure 3. In ex vivo EC, compared to in vivo, cell cycle regulator genes were overexpressed progressively in PM and OC conditions. In isolated culture of EC, NCPC overexpressed cell cycle regulators compared to EC in CPC. Immortalized ECs overexpressed 100% of cell cycle regulators. Taken together, this general evaluation showed slight activation of the cell cycle in ECs of corneas shortly after donors' death, and high activation during OC. In vitro, isolated ECs entered the cell cycle but the cycle stabilized when they reached confluence.

### Cell cycle signaling in endothelial cells

#### In vivo (reference)

**Figure 4:**
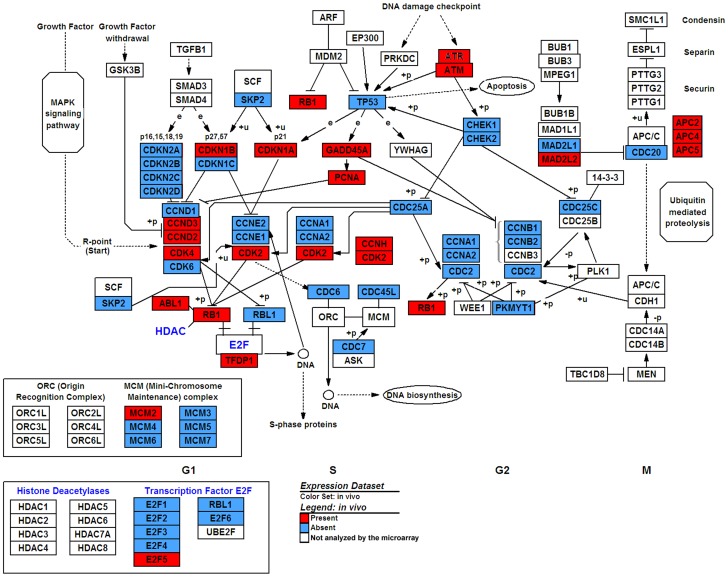
Baseline cell cycle gene expression profile in human corneal endothelial cells in vivo. Graphic representation of the presence/absence of gene expression on KEGG cell cycle pathway using GenMAPP version 2.1 (http://www.genmapp.org/).

Transcripts present and those absent in the reference group are represented in Figure 4 and detailed in Table S2 in File S1. The profile was compatible with early G1 phase arrest. For early G1 phase, CCND2 (Cyclin D2), CCND3 (Cyclin D3), CDK4 were present but not CDK6. For late G1 phase, CDK2 was present but not CCNE1 (Cyclin E1) or CCNE2 (Cyclin E2) transcripts, preventing CyclinE/CDK2 complexes from forming and entering the S phase. The presence of two CDK inhibitors CDKN1A (p21^CIP1^) and CDKN1B (p27^KIP1^) suggested inhibition of CDK2 and CDK4, preventing formation of CyclinE/CDK2 and CyclinD/CDK4 complexes. For progression from G1 to the S phase, RBL1 transcript was present but not E2F transcription factors (except for E2F5), *CDC7* (G1/S), *CDC45* (S) or MKI67 (Ki67). The CDC25A transcript required for activation of CDKs was also absent. Markers of G2 and S phase entry were absent: CCNA1 (Cyclin A1), CCNA2 (Cyclin A2), CCNB1 (Cyclin B1), CCNB2 (Cyclin B2). For the G2 to M phase, the presence of ATR and ATM transcripts suggested the activation of DNA damage checkpoints. The presence of NBN (Nibrin), GADD45A and CDKN1A (p21^CIP1^) transcripts may result in CDK2 inactivation and cell cycle arrest. The absence of M phase transcripts, such as MCM complexes (except for MCM2), confirmed the absence of cell division. Several transcripts whose proteins are involved in the large ubiquitin-mediated degradation complex were expressed: three anaphase-promoting complex (APC) subunits, three culins, and *CDC16*. Notably, no transcript of Cyclin D1, or p53 or p16^Ink4A^, was found. The five other EC types were compared to this baseline expression profile.

### Functional analysis (Fig. 5)

**Figure 5:**
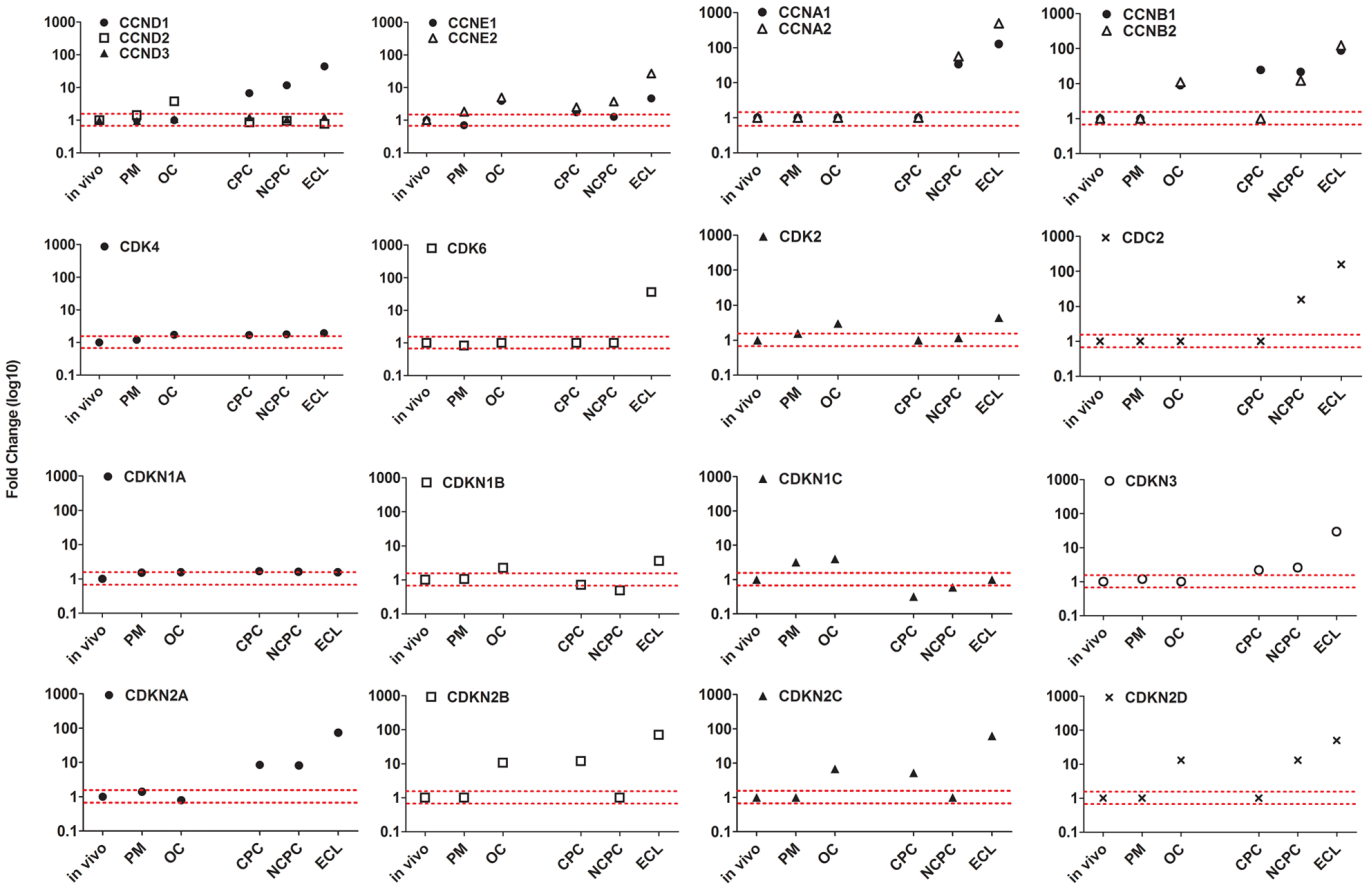
Analysis of mRNA expression of cyclins, CDKs and CKIs in endothelial cells exposed to 6 different environments. Fold-change expressions of in vivo (reference), post mortem (PM), organ culture (OC), confluent primary culture (CPC), non-confluent primary culture (NCPC), and endothelial cell line (ECL) models were represented in logarithmic scale. The red dotted lines show limits of under/overexpression (fold-change ≥1.5).

#### Expression of cyclins and cyclin-dependent kinases

For G1 to S phase, in tissue models, only cyclin D2 was overexpressed in OC. Cyclin D1 and D3 showed no significant change in any conditions. Cyclin D1, not detected in vivo, was increasingly expressed in CPC, NCPC and the ECL. Similarly, cyclin E1 and E2, not detected in vivo, were expressed in OC and primary cultures. For the formation of cyclin/CDK complexes, the level of CDK4 expression detected in vivo did not change significantly in any conditions. Only CDK6 expression, not detected in vivo, was present in the ECL. These observations suggest that the lack of expression of cyclin D1, E1, E2 and CDK6 may be responsible for EC cell-cycle arrest in G1 phase. S to G2 and M phase cyclins (A1, A2, B1, B2) and CDC2 not detected in vivo were expressed only in NCPC and the ECL. These findings were consistent with proliferation observed in both cell types. CDK2 level increased slightly only in OC and the ECL.

#### Cyclin-dependent kinase inhibitors

Notably, CDKN1A (p21) expression was unchanged in all conditions. CDKN2A (p16) was overexpressed in the three in vitro cell culture models. CDKN2B (p15), CDKN2C (p18), and CDKN2D (p19) were overexpressed in OC and also in vitro. CDKN3 significantly increased only in the three in vitro cell culture models.

### Analysis by biological model (Fig. 6)

**Figure 6:**
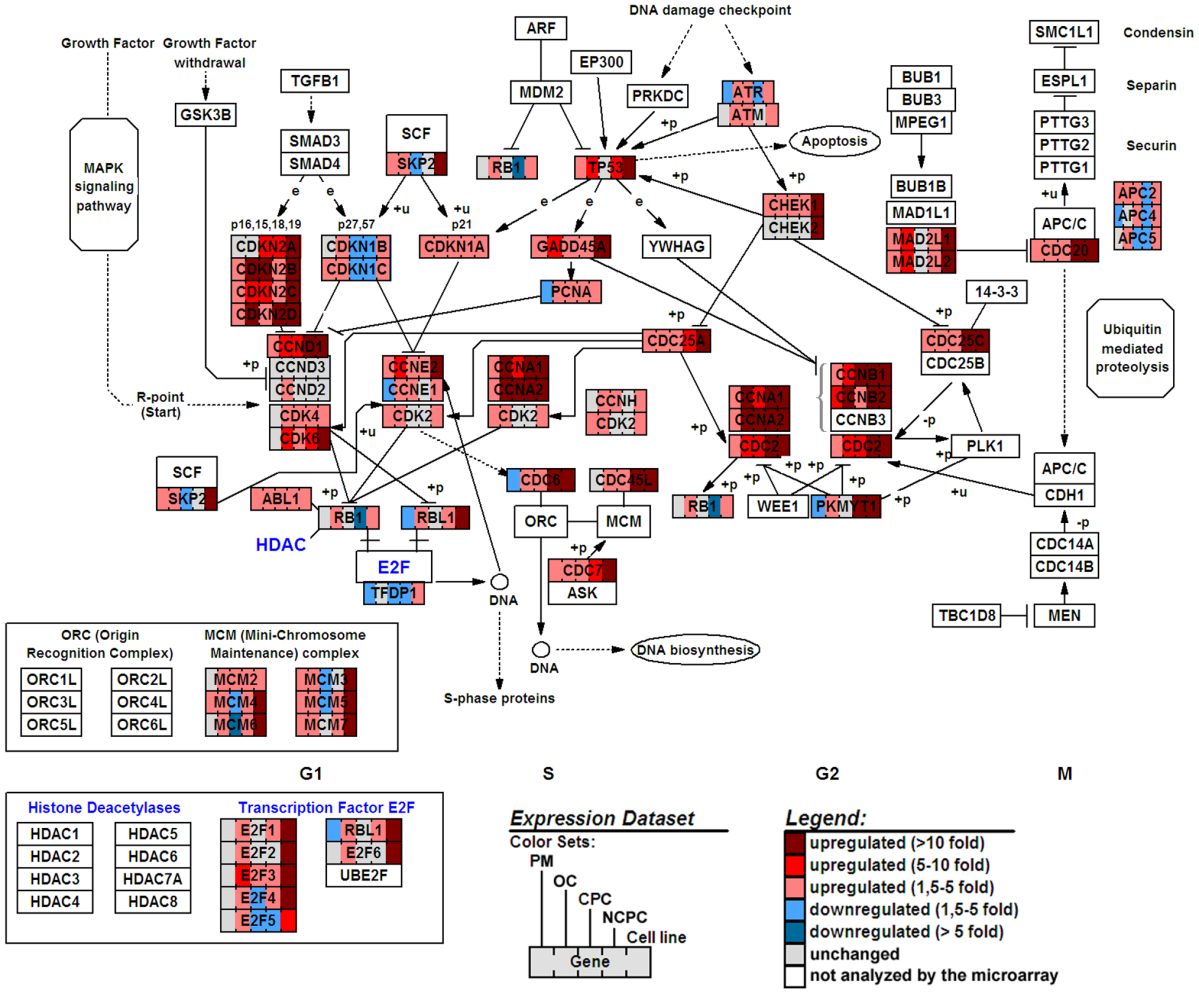
Gene map of cell cycle genes differentially expressed by human corneal endothelial cells in six biological models. Post mortem (PM), corneal organ culture (OC), in vitro confluent primary culture (CPC), in vitro non-confluent primary culture (NCPC), and immortalized endothelial cell line (ECL). Results were compared with in vivo endothelial cells. The representation of the pathway was generated by GenMAPP version 2.1 (http://www.genmapp.org/) using the cell cycle pathway provided by KEGG.

#### Post-mortem group

The total quantity of over/under-represented transcripts was lower than in other groups. The pair-wise comparison of RNA normalized expression values between in vivo and post-mortem identified 59/112 transcripts with ≥1.5-fold changes in expression (48 overexpressed, 11 underexpressed). The most over-represented transcripts were *CKS2* (+4.3 fold), *TP53* (p53) (+3.6 fold), *MAD2L2* (+3.4 fold), *CCNC* (+3.3 fold) (Table 4). Over-represented transcripts mainly comprised genes potentially associated with cell cycle arrest: *TP53* and *GADD45A*, both cyclin G, which are associated with DNA-induced damage (Cyclin G1 is associated with the p53 pathway [52]; cyclin G2 inhibits proliferation in models of ovarian [53] or gastric [54] cancers); cyclin C, which is a candidate tumor suppressor gene in osteosarcoma [55] and actively maintains quiescence in hematopoietic stem/progenitor cells [56]; *MAD2-1* and *MAD2-2*, two of the essential mitotic spindle checkpoint regulators, which have been associated with limitation of proliferation in a gastric-cancer model [57]; and p57Kip2, a strong inhibitor of several G1 phase cyclin/Cdk complexes and a negative regulator of cell proliferation [58]. In contrast, only three transcripts of pro-proliferative genes were found to be over-represented: CKS2, essential for cell cycle progression and associated with proliferation in gastric cancer [59]; *CDK7*, which functions as a CDK-activating kinase (CAK) and has also been identified as an essential component of the transcription factor TFIIH, which is involved in transcription initiation and DNA repair [60], [61], [62]; and *MCM7*, critical for cell proliferation and maintaining genome stability [63]. Other genes were absent or under-represented.

**Table 4:** Top gene expression changes.

	OVEREXPRESSION			UNDEREXPRESSION		
	Gene symbol	Gene title	Fold change	Gene symbol	Gene title	Fold change
**Post -mortem**	CKS2	CDC28 protein kinase regulatory subunit 2	4.27	ATR	Ataxia telangiectasia and Rad3 related	−2.47
	TP53	Tumor protein p53 (Li-Fraumeni syndrome)	3.56	BAX	BCL2-associated X protein	−2.10
	MAD2L2	MAD2 mitotic arrest deficient-like 2 (yeast)	3.39	CUL4A	Cullin 4A	−2.08
	CCNC	Cyclin C	3.35	CDK5R2	Cyclin-dependent kinase 5, regulatory subunit 2 (p39)	−2.05
	SERTAD1	SERTA domain containing 1	3.26	ANAPC4	Anaphase promoting complex subunit 4	−1.98
	CDKN1C	Cyclin-dependent kinase inhibitor 1C (p57, Kip2)	3.22	TFDP1	Transcription factor Dp-1	−1.80
	CCNG2	Cyclin G2	3.00	KPNA2	Karyopherin alpha 2 (RAG cohort 1, importin alpha 1)	−1.74
	MCM7	MCM7 minichromosome maintenance deficient 7 (S. cerevisiae)	2.64	PCNA	Proliferating cell nuclear antigen	−1.54
	MAD2L1	MAD2 mitotic arrest deficient-like 1 (yeast)	2.25			
	CCNG1	Cyclin G1	2.15			
	CDK7	Cyclin-dependent kinase 7 (MO15 homolog, Xenopus laevis, cdk-activating kinase)	1.99			
**OC**	DIRAS3	DEAD/H (Asp-Glu-Ala-Asp/His) box polypeptide 11 (CHL1-like helicase homolog, S. cerevisiae)	32.08			
	CCNF	Cyclin F	13.14			
	CDKN2D	Cyclin-dependent kinase inhibitor 2D (p19, inhibits CDK4)	13.05			
	CCNB2	Cyclin B2	10.95			
	CDKN2B	Cyclin-dependent kinase inhibitor 2B (p15, inhibits CDK4)	10.52			
	CCNC	Cyclin C	9.88			
	CCNB1	Cyclin B1	8.97			
	GADD45A	Growth arrest and DNA-damage-inducible, alpha	7.96			
	CUL2	Cullin 2	7.84			
	CCNT2	Cyclin T2	7.71			
	CDK5RAP3	CDK5 regulatory subunit associated protein 3	7.35			
	CDK7	Cyclin-dependent kinase 7 (MO15 homolog, Xenopus laevis, cdk-activating kinase)	7.27			
	CCNG2	Cyclin G2	6.78			
**CPC**	DIRAS3	DEAD/H (Asp-Glu-Ala-Asp/His) box polypeptide 11 (CHL1-like helicase homolog, S. cerevisiae)	43.06	UBE1	Ubiquitin-activating enzyme E1 (A1S9T and BN75 temperature sensitivity complementing)	−3.33
	BIRC5	Baculoviral IAP repeat-containing 5 (survivin)	38.92	ANAPC5	Anaphase promoting complex subunit 5	−2.49
	CCNB1	Cyclin B1	24.30	TFDP1	Transcription factor Dp-1	−2.01
	CDKN2B	Cyclin-dependent kinase inhibitor 2B (p15, inhibits CDK4)	11.83	CCNG1	Cyclin G1	−1.62
	CKS2	CDC28 protein kinase regulatory subunit 2	9.64	DDX11	DEAD/H (Asp-Glu-Ala-Asp/His) box polypeptide 11 (CHL1-like helicase homolog, S. cerevisiae)	−1.56
	CDKN2A	Cyclin-dependent kinase inhibitor 2A (melanoma, p16, inhibits CDK4)	8.51			
	CCND1	Cyclin D1	6.66			
	CCNG2	Cyclin G2	6.44			
	CDKN2C	Cyclin-dependent kinase inhibitor 2C (p18, inhibits CDK4)	5.19			
	GADD45A	Growth arrest and DNA-damage-inducible, alpha	4.51			
**NCPC**	CCNA2	Cyclin A2	56.21	RB1	Retinoblastoma 1 (including osteosarcoma)	−4.06
	BIRC5	Baculoviral IAP repeat-containing 5 (survivin)	50.85	ANAPC2	Anaphase promoting complex subunit 2	−2.78
	CCNA1	Cyclin A1	33.75	CDKN1B	Cyclin-dependent kinase inhibitor 1B (p27, Kip1)	−2.03
	CCNB1	Cyclin B1	21.65	ATR	Ataxia telangiectasia and Rad3 related	−1.99
	CDC2	Cell division cycle 2, G1 to S and G2 to M	15.54	ANAPC4	Anaphase promoting complex subunit 4	−1.81
	BRCA2	Breast cancer 2, early onset	14.96	TFDP1	Transcription factor Dp-1	−1.80
	CDC20	Cell division cycle 20 homolog (S. cerevisiae)	14.53	CUL4A	Cullin 4A	−1.62
	CDC37	Cell division cycle 37 homolog (S. cerevisiae)	13.89	ANAPC5	Anaphase promoting complex subunit 5	−1.51
	CDKN2D	Cyclin-dependent kinase inhibitor 2D (p19, inhibits CDK4)	13.10			
	DIRAS3	DEAD/H (Asp-Glu-Ala-Asp/His) box polypeptide 11 (CHL1-like helicase homolog, S. cerevisiae)	12.81			
**ECL**	CCNA2	Cyclin A2	492.07			
	CDC2	Cell division cycle 2, G1 to S and G2 to M	156.81			
	CDC6	Cell division cycle 6 homolog (S. cerevisiae)	135.25			
	CCNA1	Cyclin A1	127.09			
	CCNB2	Cyclin B2	123.42			
	BIRC5	Baculoviral IAP repeat-containing 5 (survivin)	99.46			
	CCNB1	Cyclin B1	87.46			
	CDC25C	Cell division cycle 25 homolog C (S. pombe)	76.85			
	CDKN2A	Cyclin-dependent kinase inhibitor 2A (melanoma, p16, inhibits CDK4)	73.44			
	PKMYT1	Protein kinase, membrane associated tyrosine/threonine 1	70.07			

#### Organ culture group

The pair-wise comparison of RNA normalized expression values between in vivo and OC identified 58/112 transcripts with >1.5-fold changes in expression (all overexpressed). Among the transcripts with the largest fold change in expression (Table 4), the most interesting observation was that several over-represented transcripts, not previously reported, encoded proteins implicated in cell cycle arrest. The most over-represented transcripts were *DIRAS 3*(ARH1) (+32.1 fold), *CCNF* (cyclin F) (+13.1 fold), *CDKN2D* (p19^Ink4d^), (+13.1 fold), *CDKN2B* (p15^Ink4b^) (+11.8 fold). Notably, multiple transcripts of proteins implied in cell cycle arrest in response to DNA damage were over-represented. Several other transcripts encoding pro-proliferative proteins were over-represented: *CCNE1*, *CCNE2*, *CCND2*, *CDK2*, *CDK4*, *E2F2*.

#### Confluent primary culture

The pair-wise comparison of RNA normalized expression values between in vivo and confluent primary cultures identified 32 transcripts with ≥1.5 fold changes in expression (23 overexpressed, 9 underexpressed). Among the transcripts with the largest fold changes in expression (Table 4) were six of the top 10 encoded proteins implicated in cell cycle arrest (DNA-damaged induced or CKI) and four of the top 10 encoded proteins implicated in proliferation and cell survival. The four most over-represented transcripts were *DIRAS3* (+43.1 fold), *BIRC5* (Survivin) (+38.9 fold), *CCNB1* (cyclin B1)(+24.3 fold), and *CDKN2B* (p15^Ink4b^) (+11.8 fold). The most under-represented transcripts were *UBE1* (−3.3 fold), *ANAPC5* (−2.5 fold), *TFDP1* (−2.0 fold), *E2F5* (−1.9 fold). Confluent primary culture also exhibited overexpression of transcripts of the proliferation marker *Ki67*, underexpression of *CDKN1B* (p27^KIP1^) but overexpression of three other CKIs (*CDKN2B* (p15^Ink4b^), *CDKN2A* (p16^Ink4a^), *CDKN2C* (p18^Ink4c^)), the associated cdk2 inhibitor transcript *CDKN3*, and p21^CIP1^associated transcript *BCCIP*.

#### Non-confluent primary culture

Confluent and non-confluent primary cultures exhibited similar profiles. The pair-wise comparison of RNA normalized expression values between in vivo and confluent primary cultures identified 36 transcripts with ≥1.5 fold changes in expression (22 overexpressed, 14 underexpressed). Among the transcripts with the largest change in expression (Table 4) were three of the top 10 encoded proteins implicated in cell cycle progression and four encoded proteins implicated in cell cycle inhibition. The four most overrepresented transcripts were *BIRC5* (survivin) (+50.9 fold), *CCNB1* (cyclin B1) (+21.7 fold), *BRCA2* (+15.0 fold), and *CDC20* (+14.5 fold). The most underrepresented transcripts were *CUL5* (−8.8 fold), *RB1* (−4.1 fold), *CDK5R2* (−3.3 fold), and *DDX11* (−2.9 fold). Transcripts of proliferation markers (*Ki67* and *MCM2*) were overrepresented. Non-confluent culture overexpressed fewer CKIs than confluent culture.

#### Cell line

The proliferative cell line exhibited a completely different transcriptional profile. The pair-wise comparison of RNA normalized expression values between in vivo and cell line identified 85 transcripts with ≥1.5 fold changes in expression (all overexpressed). Among the transcripts with the largest fold change in expression (Table 4), the most interesting observation was that several transcripts overrepresented in the cell line encoded proteins implicated in cell proliferation. The most overrepresented transcripts were *CCNA2* (+492.1 fold), *CDC2* (+156.8 fold), *CDC6* (+135.3 fold), *CCNB2* (+123.4 fold). The cell line exhibited expression changes in several groups of genes; and interestingly, numerous transcripts of cell cycle negative regulators were overrepresented too. Note that *DIRAS3*, overrepresented in OC and in both in vitro cultures, was not found in the ECL.

### Real-time PCR confirmation of microarrays

**Figure 7:**
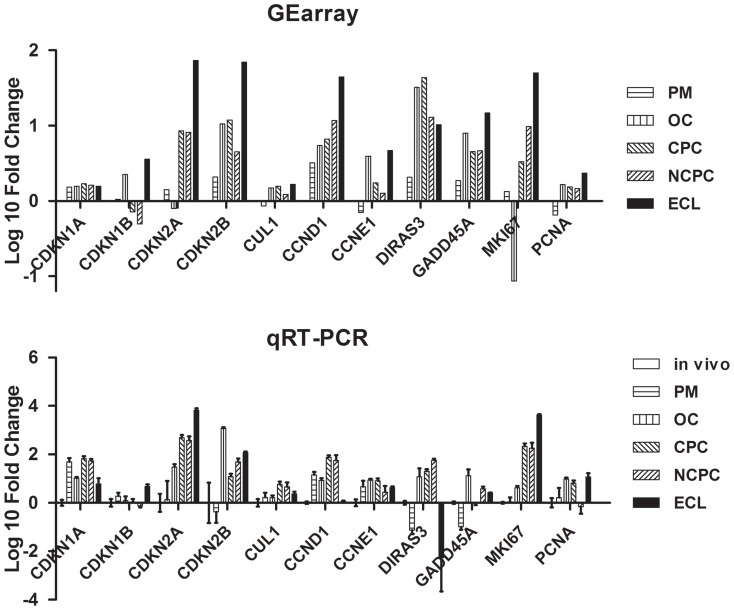
Differentially expressed genes by endothelial cells of the six different biological models, confirmed by real-time PCR. RNA (300 ng) was characterized using qRT-PCR profiling kit on a subset of 11 genes. Corresponding microarray results are shown.

A subset of 11 transcripts, over- or under-represented in microarrays depending on EC type, was tested using qRT-PCR. Differential gene expression was consistent between the two independent methods of analysis, and the level of expression of the 11 genes changed in the same direction, although there were quantitative differences between the results of gene array analysis and of quantitative real-time PCR (Fig. 7). Spearman's correlation coefficient (rho) between microarray and qRT-PCR results was 0.8218 (p<0.001), confirming a very high level of agreement.

## Discussion

In the present study, gene array technology was used to examine differential cell cycle gene expression in human corneal ECs that exhibit different proliferation status. A focused cDNA array was chosen. We identified several differentially expressed genes, especially those implicated in G1-phase arrest and in DNA damage-induced cell cycle arrest in other cell types. While it is beyond the scope of this report to highlight the potential significance of each altered gene, some are of unique interest and warrant specific mention. Although less sensitive than SAGE, SADE or the most recent RNA-sequencing techniques, microarrays enable focused assessment of gene expression in a given tissue. In Gottsh, 10% of SAGE tags were devoted to cell growth and regulation, so it was likely that specific arrays could identify specific variations in cell cycle-related transcripts in ECs. Focused analysis and transcript classification confirmed the logical evolution of the profiles from in vivo to ECL, or from the least to the most proliferative status.

### Study limitations

Despite a high level of agreement between qRT-PCR and microarrays, the changes in expression detected by microarray analysis were generally smaller than those derived by qRT-PCR as previously described [37]. This may be due to the different detection ranges of these two techniques. It has been demonstrated that microarrays tend to have a low dynamic range, which could lead to under-representation of changes in gene expression, whereas qRT-PCR has a high dynamic range [64]. This may also explain why *CCND1*, *CDNK1A*, and *MKI67*, or changes thereto, were not detected by microarray in certain groups but were detected by real-time PCR.

Ages in the in vivo group were lower than in the other groups because keratoconus patients are younger than corneal donors. In most European countries and particularly in France, where no upper age limit exists for corneal donation, mean donor age is 72 years [14]. The corneas studied in this paper are thus close in age to those routinely grafted, making it difficult to distinguish differences in transcription levels due respectively to donor aging and to environment (post-mortem stress, OC, in vitro cell culture). Donor age was significantly higher than in other studies, and our results are consequently difficult to compare, since a systemic decrease in transcription and translation with age has been described [65].

The in vivo group comprised only central ECs, whereas the other groups involved the whole endothelium, in which substantial differences in proliferative capacity between center and periphery were demonstrated [22], [23]. This likely explains the specific profile found in the in vivo group, where most transcripts were associated with cell cycle arrest and more specifically growth arrest following DNA damage; and the presence in the other groups of both cell cycle arrest and pro-proliferative signatures, because neither central nor peripheral cells were separated.

Confluent and non-confluent primary cultures were grown from independent donors.

Microarrays are limited by the finite number of oligonucleotide sequences on a chip, and are not able to identify the expression of novel genes, contrary to SAGE or genome-wide assays. Nevertheless the 112 genes contained in the present chip covered all the cell cycle genes already described and numerous others never previously studied.

Protein expression remains to be demonstrated and quantified on selected candidates to validate microarrays and qRT-PCR findings, given that post-transcriptional modifications are very likely to occur, increasing the complexity of cell cycle controls. This will be our next step, using immunolabeling that we recently optimized for en face observation of intact ECs on flat mounted corneas [66].

### Baseline transcription profile in vivo

For the first time, the cell cycle-related transcription profile of human ECs very close to in vivo physiological condition has been described using normal ECs collected immediately after explantation during a corneal graft. This profile is consistent with the previously known proliferative status determined using study of proteins involved in cell cycle regulation performed with donor corneas stored for 24–36 hours at 4°C [67], but provides much complementary information. We found multiple transcripts compatible with early G1 phase arrest of the cell cycle. *GADD45A*, *Nibrin (NBN* or *NBS1)*, *ATM* and *ATR*, four transcripts associated with DNA damage-induced cell cycle arrest, have been identified. These results are consistent with the recent findings of Joyce et al. [68] using PCR-based microarray (and Western blots for GADD45A and ATM) and with the previously described nuclear oxidative DNA damage in ECs of donors aged over 50 years, with a predominance of lesions in the central corneal area [65]. These lesions are likely induced by cumulative low-dose exposure to UV radiation throughout life (the major part of UV wavelengths being attenuated by the epithelial and stromal layers) and may be modulated by frequent temperature variations of the cornea. At chromosome level, an age-related accumulation of errors was described in human ECs, with an age-related increase in the relative number of polyploid cells with >4N DNA content and cells with multiple nuclei in otherwise healthy human corneal endothelium [69], [70]. Similar increases in DNA content were observed in adult endothelium in response to injury [71].

Several actors in the ubiquitin/proteasome complex implicated in the regulation of CDK/Cyclin and CKI have also been found, confirming that ECs actively regulate their non-proliferative profile and are not excluded from the cell cycle. Our results differ slightly from the literature regarding three transcripts: CCND1, TP53 and CDN2A. We found no expression of CCND1 or CDKN2A in microarrays, only in qRT PCR (see above for technical discussion). Until now, the three cyclin-D isoforms had not been studied separately in human ECs [67], [72]. The CCND1 protein (cyclin D1) had been described only in in vitro rat corneal endothelial cells [73]. Cyclin D isoform may have redundant functions and is not distributed equally in all tissues [74]. Nevertheless, the absence of cyclin D1 may be associated with the non-proliferative status of ECs in vivo, as overexpression was observed in OC and in the three types of cell culture. Notably, it has recently been demonstrated that the forced overexpression of CDK4/Cyclin D1 in human ECs led to the establishment of functioning ECL with high growth potential [75]. *CDKN2A* (p16^Ink4a^) was not found either, although expression of the corresponding protein in EC nuclei has already been described, with expression increasing with donor age [24], [72], [76]. This difference is unlikely explained by the donor age in our experiments because *CDKN2A* was not more strongly expressed in the OC group, although donors were older than in the in vivo group. The difference in tissue origin and storage method may be partly responsible, p16^Ink4a^ having been described in post-mortem cornea with short-term cold storage [24], [72], [76]. Conversely we found *CDKN2A* in cell cultures, as already described [24]. *TP53* transcript was not found either in microarrays (not tested with qRT-PCR), although the protein has previously been described in human donor cornea after short-term cold storage [72], [77], [78]. The difference could be explained by the absence of post-mortem and storage-induced stress in our in vivo samples. Moreover, p53 transcript was one of the most overrepresented in the post-mortem group. Lastly, we found only one of the EF2 transcription factors, E2F5, whose particular role in the early G1 phase has already been described [79].

### Donor cornea

High post-mortem stress is liable to induce profound changes in gene transcription. Unsurprisingly, the level of change in our study remained low, indicating a global decrease in cell metabolism. Nevertheless, the well-known long survival of corneal EC after donor death (successful grafts with corneas retrieved up to 72 hours post death are regularly observed, and longer post-mortem times have also been described more anecdotally) explains the possibility of observing significant transcriptional changes. DNA damage appears to activate cell cycle arrest mechanisms through overexpression of several genes involved in the p53-dependent or -independent pathways. Interestingly, culin4A, implicated in the limitation of DNA-damage repair capacity in UV-induced skin cancers [80], is under-represented in this group, and this may correspond to a response adopted to increase cellular DNA repair capacity.

### Organ-cultured corneas

Genomic study of OC corneas is also original because only corneas stored at 4°C had previously been studied. Routine OC with conventional medium (MEM+2% FCS) is the main method used for cornea storage in Europe. By promoting cell metabolism in cell culture-derived medium at 31–34°C (as per eye bank protocol) it allows storage for up to 5 weeks, unlike the current 5–6 days allowed by the cold storage at 4°C common in the USA. OC is not known to promote EC proliferation, although it facilitates endothelial wound healing by migration of cells in the vicinity of the local defect in the endothelial layer [13]. Only OC with 8% FCS, a method that remains an exception [14], seems likely to promote cell mitosis [15]. It was thus important to determine whether routine OC induces significant transcriptional profile changes liable to reveal the stimulation of certain ECs with residual proliferative capacity. In our samples, the majority of the transcripts are associated with cell cycle arrest mechanisms, but several others were implicated in G1/S transition, suggesting that ECs did not behave homogeneously in OC. The percentage of cells progressing in the cell cycle remains to be determined, but similar focused microarrays were sensitive enough to detect as little as 10% contamination from a specific cell subpopulation [81].

Up to now, the role of three CKIs (p21^Cip1^, p27^Kip1^, and p16^Ink4a^) has been demonstrated. We have highlighted the probable role of the third CIP/KIP p57^Kip2^ and the three other INK4 family members p15^Ink4b^, p18^Ink4c^, p19^Ink4d^, whose transcripts were found to be overexpressed. The absence of p16^Ink4a^ transcript has been discussed above. Our results suggest that cell cycle inhibition is redundantly controlled in OC. Interestingly, other transcripts associated with cell cycle arrest were over-represented, especially DIRAS3 (also over-represented in primary cell cultures but absent in the ECL). DIRAS3, also known as A ras homologue member 1 (ARHI) is a maternally imprinted human tumor suppressor gene that encodes a small G protein with 50–60% aminoacide homology to Rap and Ras. It has been extensively studied in various cancer types. In ovarian and breast cancer, reexpression of ARH1 inhibits cancer cell growth in vitro and in vivo, induces p21^WAF1^ and downregulates cyclin D1 promoter activity [82]. In pancreatic cancer, ARH1 blocks cell progression in cell cycle phase G1, and increases p21^Cip1^ through the accumulation of p53 protein. ARHI enhances expression of p27^Kip1^ through inhibition of PI-3K/AKT signaling [83]. Note that the Pi-3K/AKT signaling pathway has been described in rabbit ECs in response to FGF-2 stimulation in vitro [84]. However, *DIRAS3* was not found in the CorneaNet database nor in the recent study by Joyce et al [68]. Besides *DIRAS3*, several transcripts whose proteins are involved in cell cycle arrest and DNA damage repair in response to DNA damage were described in OC: *GADD45A*, *NBN*, *ATM* and *ATR* already present in the in vivo group, associated with *RAD1*, *RAD9A*, *TP53*, *BCRA1*, and *CCNG1*.

The role of CUL4 should also be further investigated because of its particular relevance in controlling DNA repair capacities. We found its transcript in in vivo EC, underexpressed in the primary cultures, and over-represented in OC and the ECL. The CUL4A-E3 ligase is involved in the ubiquitination of various protein substrates around the site of UV-induced DNA damage [85]. It is associated with UV-damaged DNA-binding protein 2 that has been described as highly expressed in human EC [86]. By analogy with descriptions made in UV-induced skin carcinogenesis in mice, in which CUL4 ubiquitin ligase restricts cellular repair capacity [80], overexpression of CUL4 in OC or the ECL may suggest that ECs do not operate at their full DNA repair potential, and highlight the potential increase in cell repair proficiency by pharmacological CUL4A inhibition.

### In vitro primary cell cultures

Confluent and non-confluent cultures did not differ fundamentally in profile. Like ECs from OC corneas, they exhibited cell cycle arrest-associated transcripts, be they CKIs, cell cycle checkpoints, or DNA damage-induced. Conversely, they also logically expressed transcripts associated with proliferation such as G1/S and G2/M cyclins, or proliferation markers. One of the most overexpressed transcripts was that of the anti-apoptotic protein survivin, which may be stimulated by cell culture-associated stress. Interestingly, the CKI p27^KIP1^ was found to be under-represented, although it was associated with mitotic inhibition induced by cell-cell contact in neonatal rat ECs [87], [88]. This result suggests that contact inhibition in confluent culture may be less efficient than in vivo, and/or that the mechanism in humans may involve other CKIs than only p27^Kip1^.

### Cell line

The SV40LT transformed ECL overexpressed almost all cell cycle-related genes in the microarray. The most up-regulated genes were logically associated with cell cycle progression and were consistent with the mechanisms of SV40LT transformation [89]: CDK/cyclin of the G1/S and G2/M transitions, E2F transcription factors, proliferation markers and MCM replication complex, with *CCNA2* and *CDC2* (both G2/M) being the two most over-represented. Conversely, cycle inhibitors (CKIs, checkpoints, DNA damage-induced inhibitors) were also strongly over-represented. A first hypothesis for the double profile relies on good contact inhibition of the cell line that, despite high proliferative capacity, strictly grows in a monolayer until it reaches very high cell density. A second explanation could be the existence of a double population in the cell line, with opposite differentiation and proliferation profiles [16], [19]. High expression of anti-apoptotic proteins is common in immortalized cells, especially IAPs [90]. Notably, the absence of overexpression of *DIRAS3* in those proliferative cells suggests it plays a role in mitosis inhibition in non-transformed ECs, where it is always present.

In conclusion, cell cycle-specific cDNA arrays identified several candidate genes that may actively repress EC proliferation in humans and thus help us understand the premature senescence in human ECs previously described [8], [23], [91]. These genes are thus a useful resource for further mechanistic studies. Several of them could be targets for triggering and controlling cell proliferation, with a view to developing a cell therapy for corneal endothelial dystrophies.

## Supporting Information

File S1 **Supporting tables. Table S1.** Functional classification by cell cycle phase (using DAVID version 6.7, 2013 (http://david.abcc.ncifcrf.gov/)) of the 62/112 transcripts obtained with the Oligo GEArray Human cell cycle Microarray. 40/112 transcripts were not classified in this table. **Table S2.** Baseline gene expression profile in human corneal endothelial cells in vivo. Highlighted in gray: transcripts that remained absent in all six experimental conditions.
(DOC)
