# Supplementary material for: Microarray Analysis of Cell Cycle Gene Expression in Adult Human Corneal Endothelial Cells
Source: PLoS One. 2014 Apr 18;9(4):e94349. doi: 10.1371/journal.pone.0094349 (PMC3991635; doi:10.1371/journal.pone.0094349)
Supplement: File S1 — Supporting tables. Table S1. Functional classification by cell cycle phase (using DAVID version 6.7, 2013 (http://david.abcc.ncifcrf.gov/)) of the 62/112 transcripts obtained with the Oligo GEArray Human cell cycle Microarray. 40/112 transcripts were not classified in this table. Table S2. Baseline gene expression profile in human corneal endothelial cells in vivo. Highlighted in gray: transcripts that remained absent in all six experimental conditions. (DOC) [file pone.0094349.s001.doc]

**Table S1 (supporting information).** Functional classification by cell cycle phase (using DAVID version 6.7, 2013 (<http://david.abcc.ncifcrf.gov/>)) of the 62/112 transcripts obtained with the Oligo GEArray Human cell cycle Microarray. 40/112 transcripts were not classified in this table.

| **Cell cycle process** | **GO terms** | **GEarray Entrez Gene Identification** |
| --- | --- | --- |
| G1 phase | GO:0000080 | CDK2 (1017), CDC6 (990), CDC25C (995), CDK6 (1021), CDKN1C (1028), E2F1 (1869) |
| S phase | GO:0000084 | DDX11 (1663), ABL1 (25) |
| G2 phase | GO:0000085 | GTSE1 (51512), KPNA2 (3838) |
| M phase | GO:0000087 | CDC25A (993), BIRC5 (332), CDK2 (1017), DDX11 (1663), CCNB1 (891), ANAPC4 (29945), ANAPC5 (51433), CCNA2 (890), CDC2 (983), MAD2L1 (4085), MAD2L2 (10459), CDC6 (990), CDC25C (995), CCNF (899), CCNG1 (900), CCNG2 (901), CDC20 (991), CDC16 (8881), CCNA1 (8900), PKMYT1 (9088), CCNB2 (9133), KNTC1 (9735), ANAPC2 (29882) |
| G1/S transition | GO:0000082, GO:0031575 | CDKN1A (1026), CDKN2B (1030), NBN (4683), CDC25A (993), GTSE1 (51512), CCND1 (595), BCL2 (596), CCND2 (894), CCNE1 (898), CDC34 (997), CDK4 (1019), CDKN1B (1027), CDKN2A (1029), CDKN2C (1031), CDKN2D (1032), CDKN3 (1033), E2F4 (1874), E2F6 (1876), MNAT1 (4331), RB1 (5925), RBBP8 (5932), SKP2 (6502), CUL5 (8065), CDC7 (8317), CUL4A (8451), CUL3 (8452), CUL2 (8453), CUL1 (8454), HUS1 (3364), TP53 (7157) |
| G2/M transition | GO:0000086, GO:0031576 | BIRC5 (332), CDKN1A (1026), CDKN2B (1030), NBN (4683), CDK2 (1017), DDX11 (1663), CCNB1 (891), ANAPC4 (29945), ANAPC5 (51433), CCNA2 (890), CDC2 (983), CHEK1 (1111), GADD45A (1647), DNM2 (1785), BRCA1 (672) |
| M/G1 (Mitotic spindle checkpoint) | GO:0007094 | BIRC5 (332), MAD2L1 (4085 ), MAD2L2 (10459), ATM (472) |

**Table S2. (supporting information).** Baseline gene expression profile in human corneal endothelial cells in vivo. Highlighted in gray: transcripts that remained absent in all six experimental conditions.

| ***Position*** | ***Symbol*** | ***Description*** | **genebank No.** | **A**bsent/**P**resent |
| --- | --- | --- | --- | --- |
| 2 | ABL1 | V-abl Abelson murine leukemia viral oncogene homolog 1 | [NM_005157](http://www.ncbi.nlm.nih.gov/nuccore/62362413) | **P** |
| 3 | ANAPC2 | Anaphase promoting complex subunit 2 | [NM_013366](http://www.ncbi.nlm.nih.gov/nuccore/41327747) | **P** |
| 4 | ANAPC4 | Anaphase promoting complex subunit 4 | [NM_013367](http://www.ncbi.nlm.nih.gov/nuccore/41327748) | **P** |
| 5 | ANAPC5 | Anaphase promoting complex subunit 5 | [NM_016237](http://www.ncbi.nlm.nih.gov/nuccore/213385321) | **P** |
| 6 | DIRAS3 | DIRAS family, GTP-binding RAS-like 3 | [NM_004675](http://www.ncbi.nlm.nih.gov/nuccore/58530880) | **A** |
| 7 | ATM | Ataxia telangiectasia mutated (includes complementation groups A, C and D) | [NM_000051](http://www.ncbi.nlm.nih.gov/nuccore/71902539) | **P** |
| 8 | ATR | Ataxia telangiectasia and Rad3 related | [NM_001184](http://www.ncbi.nlm.nih.gov/nuccore/157266316) | **P** |
| 9 | BAX | BCL2-associated X protein | [NM_004324](http://www.ncbi.nlm.nih.gov/nuccore/34335114) | **P** |
| 10 | BCCIP | BRCA2 and CDKN1A interacting protein | [NM_016567](http://www.ncbi.nlm.nih.gov/nuccore/169790844) | **A** |
| 11 | BCL2 | B-cell CLL/lymphoma 2 | [NM_000633](http://www.ncbi.nlm.nih.gov/nuccore/72198188) | **A** |
| 12 | BIRC5 | Baculoviral IAP repeat-containing 5 (survivin) | [NM_001168](http://www.ncbi.nlm.nih.gov/nuccore/59859877) | **A** |
| 13 | BRCA1 | Breast cancer 1, early onset | [NM_007294](http://www.ncbi.nlm.nih.gov/nuccore/237757283) | **A** |
| 14 | BRCA2 | Breast cancer 2, early onset | [NM_000059](http://www.ncbi.nlm.nih.gov/nuccore/119395733) | **A** |
| 15 | CCNA1 | Cyclin A1 | [NM_003914](http://www.ncbi.nlm.nih.gov/nuccore/161377466) | **A** |
| 16 | CCNA2 | Cyclin A2 | [NM_001237](http://www.ncbi.nlm.nih.gov/nuccore/166197663) | **A** |
| 17 | CCNB1 | Cyclin B1 | [NM_031966](http://www.ncbi.nlm.nih.gov/nuccore/34304372) | **A** |
| 18 | CCNB2 | Cyclin B2 | [NM_004701](http://www.ncbi.nlm.nih.gov/nuccore/10938017) | **A** |
| 19 | CCNC | Cyclin C | [NM_005190](http://www.ncbi.nlm.nih.gov/nuccore/61676090) | **A** |
| 20 | CCND1 | Cyclin D1 | [NM_053056](http://www.ncbi.nlm.nih.gov/nuccore/77628152) | **A** |
| 21 | CCND2 | Cyclin D2 | [NM_001759](http://www.ncbi.nlm.nih.gov/nuccore/209969683) | **P** |
| 22 | CCND3 | Cyclin D3 | [NM_001760](http://www.ncbi.nlm.nih.gov/nuccore/209915551) | **P** |
| 23 | CCNE1 | Cyclin E1 | [NM_001238](http://www.ncbi.nlm.nih.gov/nuccore/17318558) | **A** |
| 24 | CCNE2 | Cyclin E2 | [NM_057735](http://www.ncbi.nlm.nih.gov/nuccore/17318562) | **A** |
| 25 | CCNF | Cyclin F | [NM_001761](http://www.ncbi.nlm.nih.gov/nuccore/118572587) | **A** |
| 26 | CCNG1 | Cyclin G1 | [NM_004060](http://www.ncbi.nlm.nih.gov/nuccore/40805830) | **P** |
| 27 | CCNG2 | Cyclin G2 | [NM_004354](http://www.ncbi.nlm.nih.gov/nuccore/187608552) | **A** |
| 28 | CCNH | Cyclin H | [NM_001239](http://www.ncbi.nlm.nih.gov/nuccore/17738313) | **P** |
| 29 | CCNT1 | Cyclin T1 | [NM_001240](http://www.ncbi.nlm.nih.gov/nuccore/17978465) | **A** |
| 30 | CCNT2 | Cyclin T2 | [NM_001241](http://www.ncbi.nlm.nih.gov/nuccore/17978467) | **A** |
| 31 | CDC16 | Cell division cycle 16 homolog (S. cerevisiae) | [NM_003903](http://www.ncbi.nlm.nih.gov/nuccore/118402579) | **P** |
| 32 | CDC2 | Cell division cycle 2, G1 to S and G2 to M | [NM_001786](http://www.ncbi.nlm.nih.gov/nuccore/195927038) | **A** |
| 33 | CDC20 | Cell division cycle 20 homolog (S. cerevisiae) | [NM_001255](http://www.ncbi.nlm.nih.gov/nuccore/118402581) | **A** |
| 34 | CDC25A | Cell division cycle 25 homolog A (S. pombe) | [NM_001789](http://www.ncbi.nlm.nih.gov/nuccore/42490757) | **A** |
| 35 | CDC25C | Cell division cycle 25 homolog C (S. pombe) | [NM_001790](http://www.ncbi.nlm.nih.gov/nuccore/125625349) | **A** |
| 36 | CDC34 | Cell division cycle 34 homolog (S. cerevisiae) | [NM_004359](http://www.ncbi.nlm.nih.gov/nuccore/16357476) | **A** |
| 37 | CDC37 | Cell division cycle 37 homolog (S. cerevisiae) | [NM_007065](http://www.ncbi.nlm.nih.gov/nuccore/39995072) | **A** |
| 38 | CDC45L | CDC45 cell division cycle 45-like (S. cerevisiae) | [NM_003504](http://www.ncbi.nlm.nih.gov/nuccore/34335230) | **A** |
| 39 | CDC6 | Cell division cycle 6 homolog (S. cerevisiae) | [NM_001254](http://www.ncbi.nlm.nih.gov/nuccore/51944959) | **A** |
| 40 | CDC7 | Cell division cycle 7 homolog (S. cerevisiae) | [NM_003503](http://www.ncbi.nlm.nih.gov/nuccore/197313663) | **A** |
| 41 | CDK2 | Cyclin-dependent kinase 2 | [NM_001798](http://www.ncbi.nlm.nih.gov/nuccore/166362720) | **P** |
| 42 | CDK4 | Cyclin-dependent kinase 4 | [NM_000075](http://www.ncbi.nlm.nih.gov/nuccore/16936531) | **P** |
| 43 | CDK5R1 | Cyclin-dependent kinase 5, regulatory subunit 1 (p35) | [NM_003885](http://www.ncbi.nlm.nih.gov/nuccore/34304373) | **A** |
| 44 | CDK5R2 | Cyclin-dependent kinase 5, regulatory subunit 2 (p39) | [NM_003936](http://www.ncbi.nlm.nih.gov/nuccore/42741664) | **P** |
| 45 | CDK5RAP1 | CDK5 regulatory subunit associated protein 1 | [NM_016408](http://www.ncbi.nlm.nih.gov/nuccore/28872781) | **A** |
| 46 | CDK5RAP3 | CDK5 regulatory subunit associated protein 3 | [NM_176096](http://www.ncbi.nlm.nih.gov/nuccore/28872791) | **A** |
| 47 | CDK6 | Cyclin-dependent kinase 6 | [NM_001259](http://www.ncbi.nlm.nih.gov/nuccore/223718130) | **A** |
| 48 | CDK7 | Cyclin-dependent kinase 7 | [NM_001799](http://www.ncbi.nlm.nih.gov/nuccore/161016768) | **A** |
| 49 | CDK8 | Cyclin-dependent kinase 8 | [NM_001260](http://www.ncbi.nlm.nih.gov/nuccore/4502744) | **P** |
| 50 | CDKN1A | Cyclin-dependent kinase inhibitor 1A (p21, Cip1) | [NM_000389](http://www.ncbi.nlm.nih.gov/nuccore/169790847) | **P** |
| 51 | CDKN1B | Cyclin-dependent kinase inhibitor 1B (p27, Kip1) | [NM_004064](http://www.ncbi.nlm.nih.gov/nuccore/207113192) | **P** |
| 52 | CDKN1C | Cyclin-dependent kinase inhibitor 1C (p57, Kip2) | [NM_000076](http://www.ncbi.nlm.nih.gov/nuccore/169790897) | **A** |
| 53 | CDKN2A | Cyclin-dependent kinase inhibitor 2A (p16) | [NM_000077](http://www.ncbi.nlm.nih.gov/nuccore/47132606) | **A** |
| 54 | CDKN2B | Cyclin-dependent kinase inhibitor 2B (p15) | [NM_004936](http://www.ncbi.nlm.nih.gov/nuccore/47132608) | **A** |
| 55 | CDKN2C | Cyclin-dependent kinase inhibitor 2C (p18) | [NM_078626](http://www.ncbi.nlm.nih.gov/nuccore/89903019) | **A** |
| 56 | CDKN2D | Cyclin-dependent kinase inhibitor 2D (p19) | [NM_001800](http://www.ncbi.nlm.nih.gov/nuccore/39995074) | **A** |
| 57 | CDKN3 | Cyclin-dependent kinase inhibitor 3 | [NM_005192](http://www.ncbi.nlm.nih.gov/nuccore/195927023) | **A** |
| 58 | CHEK1 | CHK1 checkpoint homolog (S. pombe) | [NM_001274](http://www.ncbi.nlm.nih.gov/nuccore/166295191) | **A** |
| 59 | CHEK2 | CHK2 checkpoint homolog (S. pombe) | [NM_007194](http://www.ncbi.nlm.nih.gov/nuccore/54112404) | **A** |
| 60 | CKS1B | CDC28 protein kinase regulatory subunit 1B | [NM_001826](http://www.ncbi.nlm.nih.gov/nuccore/206725531) | **P** |
| 61 | CKS2 | CDC28 protein kinase regulatory subunit 2 | [NM_001827](http://www.ncbi.nlm.nih.gov/nuccore/4502858) | **P** |
| 62 | CUL1 | Cullin 1 | [NM_003592](http://www.ncbi.nlm.nih.gov/nuccore/32307160) | **P** |
| 63 | CUL2 | Cullin 2 | [NM_003591](http://www.ncbi.nlm.nih.gov/nuccore/19482173) | **A** |
| 64 | CUL3 | Cullin 3 | [NM_003590](http://www.ncbi.nlm.nih.gov/nuccore/211971005) | **A** |
| 65 | CUL4A | Cullin 4A | [NM_003589](http://www.ncbi.nlm.nih.gov/nuccore/57165422) | **P** |
| 66 | CUL5 | Cullin 5 | [NM_003478](http://www.ncbi.nlm.nih.gov/nuccore/67514034) | **P** |
| 67 | DDX11 | DEAD/H (Asp-Glu-Ala-Asp/His) box polypeptide 11 | [NM_004399](http://www.ncbi.nlm.nih.gov/nuccore/100913203) | **P** |
| 68 | DNM2 | Dynamin 2 | [NM_004945](http://www.ncbi.nlm.nih.gov/nuccore/56549118) | **A** |
| 69 | E2F1 | E2F transcription factor 1 | [NM_005225](http://www.ncbi.nlm.nih.gov/nuccore/168480109) | **A** |
| 70 | E2F2 | E2F transcription factor 2 | [NM_004091](http://www.ncbi.nlm.nih.gov/nuccore/34485718) | **A** |
| 71 | E2F3 | E2F transcription factor 3 | [NM_001949](http://www.ncbi.nlm.nih.gov/nuccore/168480112) | **A** |
| 72 | E2F4 | E2F transcription factor 4, p107/p130-binding | [NM_001950](http://www.ncbi.nlm.nih.gov/nuccore/44829053) | **A** |
| 73 | E2F5 | E2F transcription factor 5, p130-binding | [NM_001951](http://www.ncbi.nlm.nih.gov/nuccore/134142810) | **P** |
| 74 | E2F6 | E2F transcription factor 6 | [NM_198256](http://www.ncbi.nlm.nih.gov/nuccore/109637794) | **A** |
| 75 | GADD45A | Growth arrest and DNA-damage-inducible, alpha | [NM_001924](http://www.ncbi.nlm.nih.gov/nuccore/9790904) | **P** |
| 76 | GTF2H1 | General transcription factor IIH, polypeptide 1, 62kDa | [NM_005316](http://www.ncbi.nlm.nih.gov/nuccore/214831569) | **P** |
| 77 | GTSE1 | G-2 and S-phase expressed 1 | [NM_016426](http://www.ncbi.nlm.nih.gov/nuccore/253970411) | **A** |
| 78 | HERC5 | Hect domain and RLD 5 | [NM_016323](http://www.ncbi.nlm.nih.gov/nuccore/110825981) | **A** |
| 79 | HUS1 | HUS1 checkpoint homolog (S. pombe) | [NM_004507](http://www.ncbi.nlm.nih.gov/nuccore/31077213) | **A** |
| 80 | KNTC1 | Kinetochore associated 1 | [NM_014708](http://www.ncbi.nlm.nih.gov/nuccore/156071508) | **A** |
| 81 | KPNA2 | Karyopherin alpha 2 (RAG cohort 1, importin alpha 1) | [NM_002266](http://www.ncbi.nlm.nih.gov/nuccore/62388891) | **P** |
| 82 | MAD2L1 | MAD2 mitotic arrest deficient-like 1 (yeast) | [NM_002358](http://www.ncbi.nlm.nih.gov/nuccore/194688136) | **A** |
| 83 | MAD2L2 | MAD2 mitotic arrest deficient-like 2 (yeast) | [NM_006341](http://www.ncbi.nlm.nih.gov/nuccore/187960072) | **P** |
| 84 | MCM2 | MCM2 minichromosome maintenance deficient 2, mitotin (S. cerevisiae) | [NM_004526](http://www.ncbi.nlm.nih.gov/nuccore/33356546) | **P** |
| 85 | MCM3 | MCM3 minichromosome maintenance deficient 3 (S. cerevisiae) | [NM_002388](http://www.ncbi.nlm.nih.gov/nuccore/33356548) | **A** |
| 86 | MCM4 | MCM4 minichromosome maintenance deficient 4 (S. cerevisiae) | [NM_005914](http://www.ncbi.nlm.nih.gov/nuccore/33469918) | **A** |
| 87 | MCM5 | MCM5 minichromosome maintenance deficient 5, cell division cycle 46 (S. cerevisiae) | [NM_006739](http://www.ncbi.nlm.nih.gov/nuccore/143770796) | **A** |
| 88 | MCM6 | Minichromosome maintenance deficient 6 homolog (S. cerevisiae) | [NM_005915](http://www.ncbi.nlm.nih.gov/nuccore/33469920) | **A** |
| 89 | MCM7 | MCM7 minichromosome maintenance deficient 7 (S. cerevisiae) | [NM_005916](http://www.ncbi.nlm.nih.gov/nuccore/33469967) | **A** |
| 90 | MKI67 | Antigen identified by monoclonal antibody Ki-67 | [NM_002417](http://www.ncbi.nlm.nih.gov/nuccore/225543213) | **A** |
| 91 | MNAT1 | Menage a trois homolog 1, cyclin H assembly factor (Xenopus laevis) | [NM_002431](http://www.ncbi.nlm.nih.gov/nuccore/49574512) | **P** |
| 92 | MRE11A | MRE11 meiotic recombination 11 homolog A (S. cerevisiae) | [NM_005590](http://www.ncbi.nlm.nih.gov/nuccore/56550106) | **A** |
| 93 | NBN | Nibrin | [NM_002485](http://www.ncbi.nlm.nih.gov/nuccore/67189763) | **A** |
| 94 | PCNA | Proliferating cell nuclear antigen | [NM_182649](http://www.ncbi.nlm.nih.gov/nuccore/33239450) | **P** |
| 95 | PKMYT1 | Protein kinase, membrane associated tyrosine/threonine 1 | [NM_182687](http://www.ncbi.nlm.nih.gov/nuccore/33383238) | **A** |
| 96 | RAD1 | RAD1 homolog (S. pombe) | [NM_002853](http://www.ncbi.nlm.nih.gov/nuccore/76881813) | **A** |
| 97 | RAD17 | RAD17 homolog (S. pombe) | [NM_002873](http://www.ncbi.nlm.nih.gov/nuccore/4506382) | **A** |
| 98 | RAD50 | RAD50 homolog (S. cerevisiae) | [NM_005732](http://www.ncbi.nlm.nih.gov/nuccore/295442468) | **A** |
| 99 | RAD51 | RAD51 homolog (RecA homolog, E. coli) (S. cerevisiae) | [NM_002875](http://www.ncbi.nlm.nih.gov/nuccore/256017139) | **A** |
| 100 | RAD9A | RAD9 homolog A (S. pombe) | [NM_004584](http://www.ncbi.nlm.nih.gov/nuccore/19924112) | **A** |
| 101 | RB1 | Retinoblastoma 1 (including osteosarcoma) | [NM_000321](http://www.ncbi.nlm.nih.gov/nuccore/108773786) | **P** |
| 102 | RBBP8 | Retinoblastoma binding protein 8 | [NM_002894](http://www.ncbi.nlm.nih.gov/nuccore/42718012) | **P** |
| 103 | RBL1 | Retinoblastoma-like 1 (p107) | [NM_002895](http://www.ncbi.nlm.nih.gov/nuccore/34577078) | **A** |
| 104 | RBL2 | Retinoblastoma-like 2 (p130) | [NM_005611](http://www.ncbi.nlm.nih.gov/nuccore/172072596) | **A** |
| 105 | RGC32/C13orf15 | Response gene to complement 32/chromosome 13 open reading frame 15 | [NM_014059](http://www.ncbi.nlm.nih.gov/nuccore/132626810) | **A** |
| 106 | RPA3 | Replication protein A3, 14kDa | [NM_002947](http://www.ncbi.nlm.nih.gov/nuccore/52851430) | **A** |
| 107 | SERTAD1 | SERTA domain containing 1 | [NM_013376](http://www.ncbi.nlm.nih.gov/nuccore/153792059) | **A** |
| 108 | SKP2 | S-phase kinase-associated protein 2 (p45) | [NM_005983](http://www.ncbi.nlm.nih.gov/nuccore/16306594) | **A** |
| 109 | SUMO1 | SMT3 suppressor of mif two 3 homolog 1 (S. cerevisiae) | [NM_003352](http://www.ncbi.nlm.nih.gov/nuccore/54792063) | **P** |
| 110 | TFDP1 | Transcription factor Dp-1 | [NM_007111](http://www.ncbi.nlm.nih.gov/nuccore/219842208) | **P** |
| 111 | TFDP2 | Transcription factor Dp-2 (E2F dimerization partner 2) | [NM_006286](http://www.ncbi.nlm.nih.gov/nuccore/148747290) | **A** |
| 112 | TP53 | Tumor protein p53 (Li-Fraumeni syndrome) | [NM_000546](http://www.ncbi.nlm.nih.gov/nuccore/187830767) | **A** |
| 113 | UBE1 | Ubiquitin-activating enzyme E1 (A1S9T and BN75 temperature sensitivity complementing) | [NM_003334](http://www.ncbi.nlm.nih.gov/nuccore/163659922) | **P** |
